# Structural and Dynamic Properties of Flame-Retardant Phosphorylated-Polycarbonate/Polycarbonate Blends

**DOI:** 10.3390/ijms26073241

**Published:** 2025-03-31

**Authors:** Wissawat Sakulsaknimitr, Chompunut Wongsamut, Pornpen Atorngitjawat

**Affiliations:** 1 Department of Fundamental Science and Physical Education, Faculty of Science at Sriracha, Kasetsart University, Sriracha Campus, Chonburi 20230, Thailand; wissawat.s@ku.th; 2 Department of Chemistry, Faculty of Science, Burapha University, Chonburi 20131, Thailand; wchompunut@gmail.com

**Keywords:** flame retardant, dielectric relaxation, phosphorylation, dynamic fragility, polymer blend

## Abstract

The eco-friendly flame retardancy of polycarbonate (PC) was achieved by blending with phosphorylated-PC in the range of 1–5% w/w. Dynamic properties were characterized using broadband dielectric relaxation spectroscopy (DRS), while structural and thermal properties were investigated using Fourier transform infrared spectroscopy, wide-angle X-ray diffraction, small-angle X-ray scattering, differential scanning calorimetry, and thermogravimetric analysis. A reduction in the single glass transition temperature with increasing phosphorylated-PC content was observed, indicating that the blends were miscible. No crystalline phases were detected in any of the samples. The thermo-oxidative stability and UL-94 ratings of flame-retardant polycarbonates (FRPCs) improved compared to neat PC, with char residue increasing as the phosphorylated-PC content rose. DRS analysis revealed the formation of a well-defined local (β) relaxation in the FRPC samples, originating from the motion of phosphorylated branches. All samples exhibited the segmental (α) relaxation of PC chains above the glass transition temperature. The size of the cooperatively rearranging domain played a significant role in the dynamic fragility of the rigid FRPCs. Additionally, DRS analysis highlighted the presence of physical crosslinks from nanoclusters of phosphorylated polar groups, approximately 14 nm in size.

## 1. Introduction

Plastics are widely used in various applications due to their diverse properties. However, most plastics are flammable, and some have low to medium flame-retardant properties. To enhance the flame resistance of polymers, various flame-retardant additives, such as those containing bromine, chlorine, and phosphorus, are commonly employed. However, halogenated flame retardants have been found to be harmful to the environment, as well as to animals and humans. During combustion, these compounds release halogen atoms or halogen halides, which are known to be carcinogenic and toxic [1,2]. In response to these environmental concerns, phosphorus-based flame retardants have garnered increasing attention. These compounds are considered more environmentally friendly and can effectively interact with burning polymers. They create a heat barrier that blocks heat radiation, mitigates thermal feedback, and inhibits flame spread, thereby helping to enhance the material’s flame resistance while avoiding the harmful effects associated with halogenated additives [3,4,5,6,7,8,9,10]. Phosphorus-containing flame retardants (PFRs) play an essential role in reducing the flammability of materials, especially polymers, and are widely used in various industries. The mechanism of action of phosphorus-based flame retardants can be understood in both the condensed phase and the gas phase, as their behavior differs depending on the environment and the temperature conditions. In the condensed phase, phosphorus flame retardants act mainly by promoting char formation, enhancing thermal stability, cross-linking, and creating a protective layer that limits the availability of flammable gases. In the gas phase, they function by scavenging free radicals, inhibiting the combustion chain reaction, and releasing non-combustible gases that reduce the flammability of the material [11,12,13,14,15,16]. Recent research has explored their synthesis, application, and impact on material properties. Aryl phosphates are widely used as phosphorus-based flame retardants [4]. For example, bisphenol A bis(diphenyl phosphate) has been incorporated into polycarbonate/acrylonitrile butadiene styrene blends, while tris-(3-aminophenyl)-phosphate has been added to epoxy resins to enhance their flame resistance [5,6].

Polycarbonates (PCs) are known as engineering plastics, classified by high melting points as well as high thermal and hydrolytic stability. PCs are synthesized through two convenient methods. The first involves an ester exchange reaction between molten bisphenol A and an organic carbonate, such as diphenyl carbonate. The second method entails a reaction between bisphenol A and phosgene [17]. Typically, to improve the flame-retardant properties of PCs, tetrabromobisphenol A, brominated compounds, and organophosphates are added before polycondensation. However, these additives lead to lower softening temperatures, reduced mechanical properties, and the production of toxic byproducts during combustion [1,18]. Phosphorylation methods for synthesizing phosphorus-containing flame retardants have primarily focused on modifying monomers directly or incorporating these compounds as additives into polymers. While these approaches have proven effective, they often involve additional processing steps or the use of external flame retardant additives, which can alter the properties of the materials. In contrast, the present work introduces a novel approach by performing phosphorylation directly within the PC matrix itself. This method enhances the interaction between PC chains while minimizing any significant impact on the thermal and mechanical properties of the material. Therefore, phosphorylated-PC was prepared by introducing hydroxyl groups onto the PC chains via a hydrolysis process, followed by an epichlorohydrin reaction [19]. Flame-retardant PCs (FRPC) were then prepared by blending neat PC with phosphorylated-PC in various ratios. The chemical characteristics of FRPC were examined using Fourier Transform Infrared Spectroscopy (FTIR). Thermal and oxidative properties were measured using Differential Scanning Calorimetry (DSC) and Thermogravimetric Analysis (TGA). The flame retardancy was evaluated using the UL-94 test. Additionally, to elucidate the structure–property relationship, the dielectric relaxations of FRPCs will be investigated, along with other complementary studies such as Wide-Angle X-ray Diffraction (WAXD), Small-Angle X-ray Scattering (SAXS), and thermal properties.

## 2. Results and Discussion

### 2.1. Structure and Morphology

**Figure 1:**
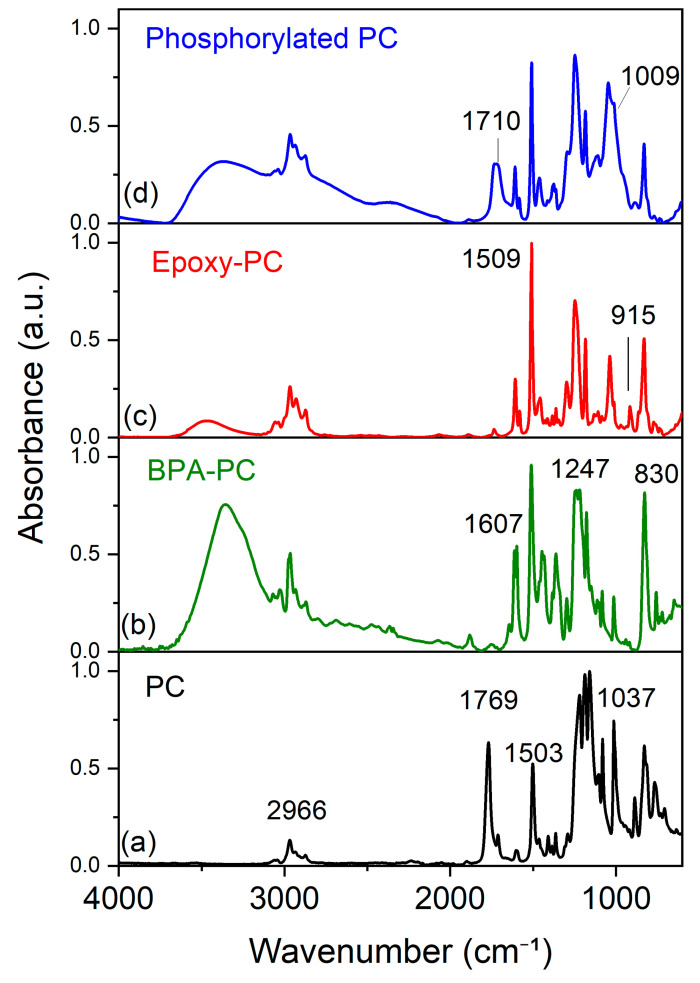
ATR-FTIR spectra of PC, BPA-PC, epoxy-PC, and phosphorylated-PC.

FTIR was used to analyze the functional groups in phosphorylated-PC. The FTIR spectrum in Figure 1a shows the spectra of PC. A characteristic carbonyl stretching (C=O) of ester linkage displays at 1769 cm^−1^. A peak at 1503 cm^−1^ is due to the vibrations of the C=C bonds in the aromatic rings. The stretching vibration of C-O was observed in the 1300–1158 cm^−1^ range. Peaks around 830 cm^−1^ arise from the bending vibration of C-H in aromatic rings. No absorption peaks corresponding to hydroxyl groups were observed in the range of 3400–3200 cm^−1^. To introduce hydroxyl groups onto the PCs, a methanolysis process was conducted, resulting in a bisphenol-A structure named BPA-PC. As shown in Figure 1b, BPA-PC exhibits a broad hydroxyl group peak in the range of 3400–3200 cm^−1^, while the carbonyl group disappears. The epoxide group in epoxy-PC, the product of BPA-PC + epichlorohydrin, is observed at 1037 and 915 cm^−1^ for C-O stretching and C-O-C vibration of epoxy rings, respectively, as seen in Figure 1c. The intensity of the hydroxyl peaks is lower than that of the C-H stretching peaks (2966 cm^−1^), indicating a reaction between BPA and epichlorohydrin. As shown in Figure 1d, the FTIR spectrum of phosphorylated-PC, the product of epoxy-PC phosphorylation, displays peaks for P-OH stretching around 1700 cm^−1^, P=O stretching around 1300 cm^−1^, P-O-C stretching around 1000 cm^−1^ [20,21,22].

**Figure 2:**
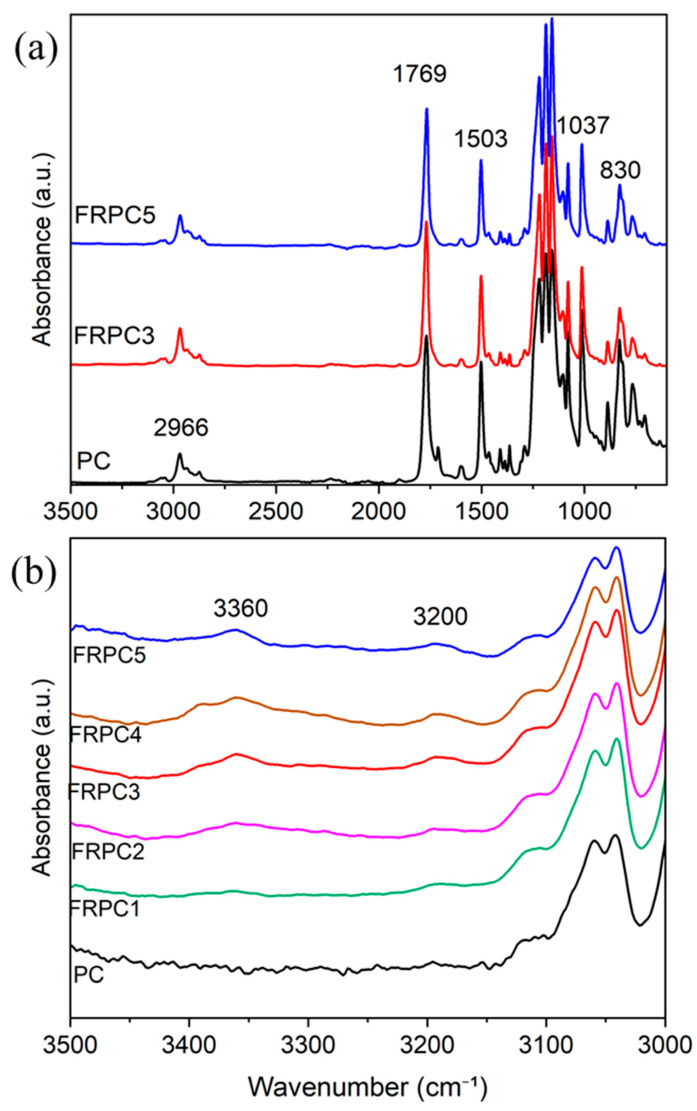
ATR-FTIR spectra of PC and FRPCs: (**a**) from 600 to 3500 cm^−1^, and (**b**) from 3000 to 3500 cm^−1^.

Figure 2a shows the FTIR spectra of FRPC3 and FRPC5 compared to neat PC. No significant differences in chemical structure were observed between PC and FRPC, as only 3% and 5% by weight of phosphorylated-PCs were blended. Figure 2b displays a portion of the spectrum between 3000–3500 cm^−1^ for neat PC and the FRPC samples. The two small, broad peaks around 3300–3400 cm^−1^ and 3200 cm^−1^ in the FRPC spectra correspond to O-H stretching vibrations, which are associated with strong and weak hydrogen bonding, respectively. These observations suggest the formation of hydrogen bonding between phosphorylated-PC and neat PC.

**Figure 3:**
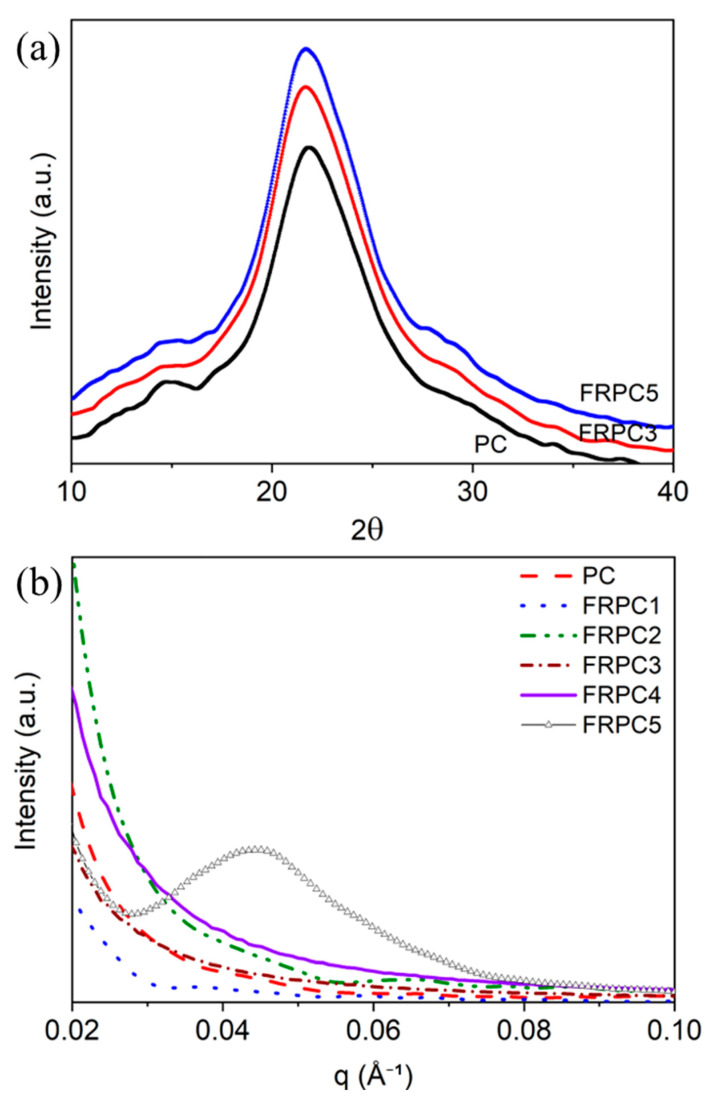
(**a**) WAXD patterns, and (**b**) Lorentz-corrected SAXS profiles of FRPCs compared to neat PC.

The degree of crystallinity of the samples was examined using WAXD. Figure 3a presents the X-ray diffraction patterns for PC and FRPCs, with FRPC3 and FRPC5 provided as examples. The absence of distinct crystalline diffraction peaks in all the samples suggests that they are completely amorphous. The phase separation of the samples was analyzed using Lorentz-corrected SAXS data. As shown in Figure 3b, no maximum scattering was observed for neat PC and FRPCs, except for FRPC5. This suggests that there is no detectable phase separation at the length scale ranging from 5 to 60 nm in PC, FRPC1, FRPC2, FRPC3, and FRPC4. Only FRPC5 displays a scattering peak at q = 0.045 Å^−1^, corresponding to a mean inter-domain spacing of approximately 14 nm, which is indicative of nanophase separation. This phase separation may result from either the relatively poor coupling between the phosphorylated-PC and the PC host polymers or the aggregation of phosphorylated polar groups in FRPC5.

### 2.2. Glass Transition Temperatures and Thermo-Oxidative Properties

**Figure 4:**
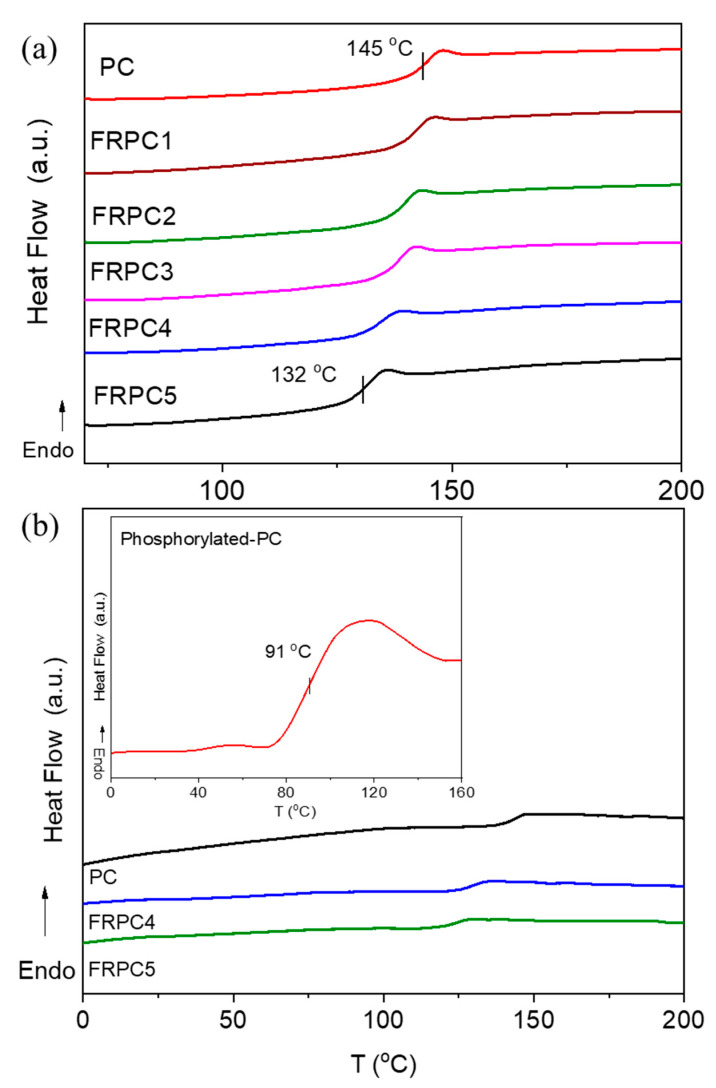
DSC thermograms of (**a**) all FRPCs compared to neat PC in the range of 60–200 °C, and (**b**) phosphorylated-PC compared to PC and FRPCs in the range of 0–160 °C.

As shown in Figure 4a,b, a single glass transition temperature (T_g_) was observed for both neat PC and all PC/phosphorylated-PC blended samples, indicating relatively homogeneous blending. The T_g_s determined directly from DSC are consistent with those estimated from dielectric relaxation spectroscopy (DRS) using VFT parameters (in Section 2.4). Figure 4b presents the T_g_ of phosphorylated-PC, approximately 91 °C, compared to neat PC and FRPC samples. The lower T_g_ of phosphorylated-PC is attributed to the reduced molecular weight of PC following hydrolysis, as confirmed by the reduction in viscosity: phosphorylated-PC (5.5 cP) is approximately five times less viscous than PC (28.4 cP). As the phosphorylated-PC content increases, the experimental T_g_ decreases slightly, from 145 °C to 132 °C, in the following order: 145, 143, 140, 138, 135, and 132 °C, respectively. However, for FRPC5, despite the aggregation of polar groups observed in the SAXS results, a single T_g_ was still detected via DSC, indicating that the blend remains homogeneous and miscible, albeit richer in phosphorylated polar groups. The DSC technique is not capable of detecting the T_g_ of the nanoaggregates in FRPCs due to the small amount of phosphorylated-PC; however, it was observed for neat phosphorylated-PC in Figure 4b at approximately 46 °C.

**Figure 5:**
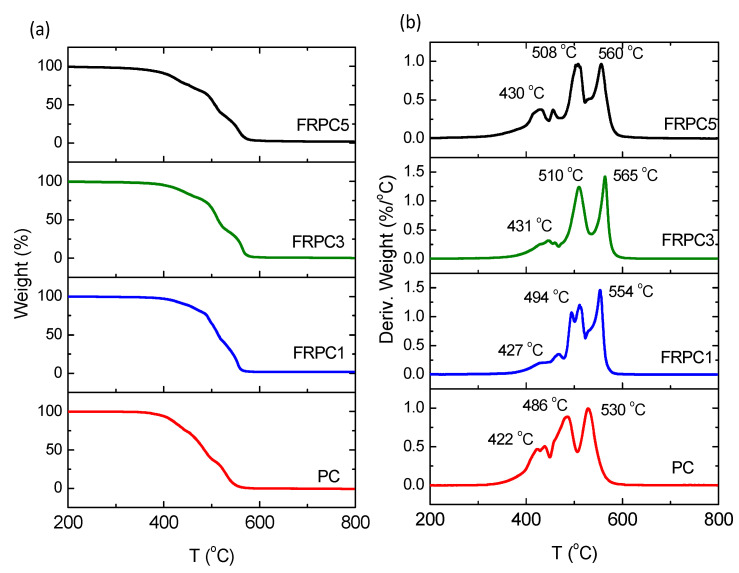
Thermo-oxidative properties of PC, FRPC1, FRPC3, and FRPC5: (**a**) % weight loss, and (**b**) derivative weight observed from TGA.

Combustion is primarily driven by the oxidation reaction between oxygen and the polymer material. The easier this oxidation occurs, the more readily ignition takes place, resulting in higher flammability. Phosphorus compounds, such as ammonium polyphosphate or organophosphates, play a critical role in promoting the formation of a carbonaceous char during the thermal degradation of polymers at elevated temperatures. This char layer acts as a protective barrier, slowing the diffusion of heat and oxygen into the material, thereby hindering further combustion [11]. The mechanism behind thermo-oxidative degradation involves the production of combustible gases and the formation of char. Consequently, the thermal degradation behavior observed in TGA can provide valuable insight into the extent of char formation. Figure 5 shows the thermal stability of neat PC and FRPC samples measured under O_2_, representing their thermo-oxidative degradation. This illustrates the temperature at which the samples can resist oxygen and how easily combustible gases are formed. As shown in Figure 5a,b, degradation begins at 422 °C for neat PC and at a higher temperature for FRPCs. The weight loss in the temperature range of 422–530 °C is associated with the release of volatile products such as phenol, bisphenol A, and carbon dioxide [1,11,13,14]. This occurs due to the chain scission and cleavage of the carbonate bonds in the polymer backbone. Figure 5b presents the derivative weight vs. temperature curve, which clearly demonstrates that neat PC is less thermo-oxidatively stable than FRPCs. The multiple peaks observed in the curve correspond to the small shoulder seen in the weight loss data. This feature is attributed to the recombination of condensed-phase radicals during the crosslinking process. It has been proposed that this crosslinking involves skeletal rearrangement, which contributes to the formation of a char layer and the creation of new chemical structures, such as benzoate esters [11,12,13,14,15]. Table 1 provides the mass of volatile products at 450, 500, and 550 °C, as well as the char residue at 650 °C for both neat PC and FRPCs. The highest levels of volatile products were observed for neat PC at all selected temperatures. At 550 °C, the addition of phosphorylated-PC flame retardants led to a decrease in volatile products. FRPC5 exhibited a higher mass of volatile products compared to the other FRPCs, which may be due to the nanophase structure of the phosphorylated polar groups, as supported by the results from SAXS and DSC. Nevertheless, the mass of volatile products and char residues at 650 °C clearly indicates that phosphorylated-PC flame retardants enhance thermo-oxidative stability.

### 2.3. Flame Retardant Property

The UL-94 test is widely used to assess the flammability of plastic materials by evaluating their behavior when exposed to a controlled flame [23]. In this test, materials are rated based on their ability to self-extinguish after ignition and the presence of dripping flames, which could potentially ignite materials located beneath the burning sample. The flame retardancy results are summarized in Table 2. The findings show that FRPC films demonstrate improved flame resistance compared to neat PC, with ratings progressing from V2 to V1. Neat PC ignites and burns completely, forming a cracked char with dripping, which indicates poor flame resistance as the molten droplets can spread the fire. In contrast, the FRPC films form hard, solid char residues, especially FRPC4 and FRPC5, suggesting a more stable and durable material that better resists combustion. However, FRPC1, FRPC2, and FRPC3 still show some burning with dripping, indicating that these formulations still have some flammability concerns. The weight loss after testing decreased with increasing phosphorylated-PC, suggesting that the phosphorylation of PC enhances its thermal stability and flame resistance. The formation of a condensed char layer, facilitated by the phosphate-based additives, plays a crucial role in improving flame retardancy by acting as a protective barrier that slows the spread of fire [11].

### 2.4. Dielectric Relaxations

**Figure 6:**
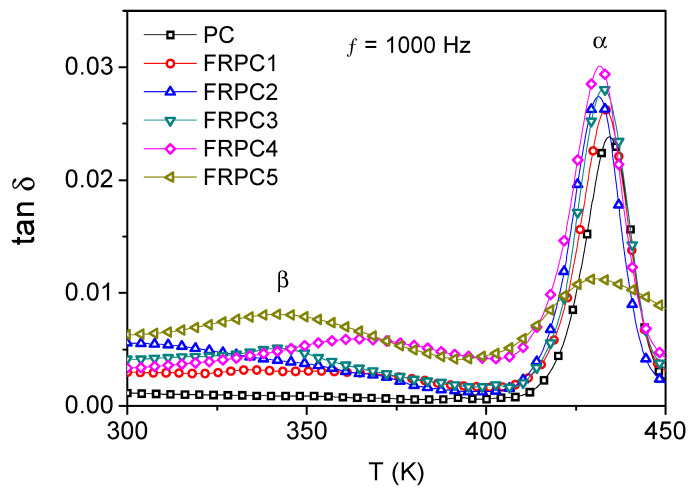
Tan δ as a function of temperatures at a frequency of 1 KHz.

Figure 6 illustrates the dielectric relaxation behavior, tanδ (tanδ = ε″ /ε′), as a function of temperatures at a frequency of 1 KHz. The FRPC samples exhibit two distinct relaxations, occurring below and above the glass transition temperature (T_g_), labeled as β and α, respectively. These relaxations correspond to the local (β) and segmental (α) relaxations. PC does not exhibit a dielectric β-process in the glassy state, while FRPC samples show a well-defined β-process, occurring within a temperature range of 65 to 100 °C in the measurement frequency window. This process is likely due to the motion of phosphorylated-PC, which forms hydrogen bonds with the carbonyl groups of PC chains. This behavior is clearly observed for FRPC 3, FRPC 4, and FRPC 5.

**Figure 7:**
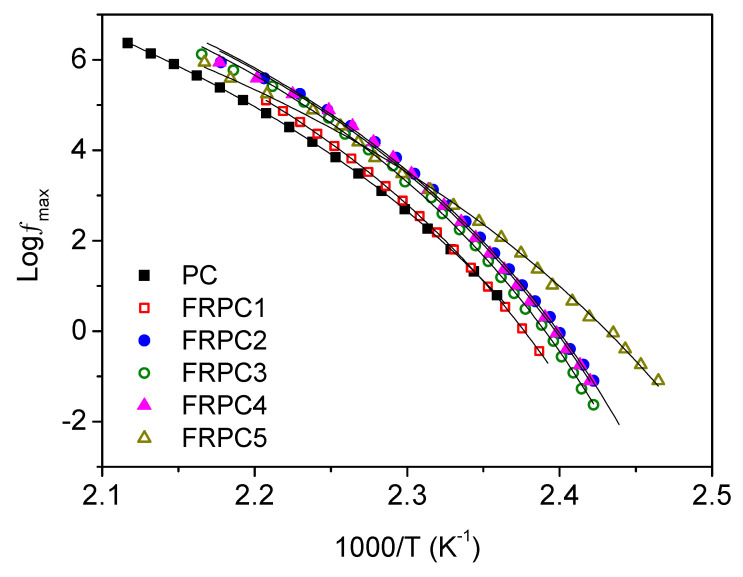
Temperature dependence of the relaxation time for the α process.

The relaxation frequencies, *f*_max_ of the α relaxation as a function of temperature for all samples, are presented in Figure 7. The α relaxations are well modeled using the Vogel–Fulcher–Tamman (VFT) relation (Equation (1)) [24], with *f*_0_ fixed at 10^–14^ s.

(1) $$f_{\max}(T)=f_{0}\exp\left(-\frac{B}{T-T_{0}}\right)$$

*f*_0_ and B are constants. *f*_0_ is the exponential prefactor or the relaxation frequency at infinite temperature, while B is related to the apparent activation energy or the fragility of the system [25]. T_0_, known as the Vogel temperature, is the threshold below which the segments become immobile. Fitting parameters B and T_0_ are provided in Table 3. The dynamic glass transition temperature (T_g_,_DRS_) can be determined from Equation (2) [26] and is provided in Table 3. The T_g_,_DRS_ is very close to T_g_,_DSC_.

(2) $$T_{g,DRS}=\frac{B}{\ln(2\pi f_{0})}+T_{0}$$

The fragility of a glass former (F), as defined by Equation (3) [26], was used to determine the cooperativity of systems, which correlates with the degree of intermolecular coupling. Dynamic fragility describes how the dynamics of the system change as it approaches the glass transition temperature. A greater fragility indicates a highly cooperative system with stronger intermolecular coupling, while a lower fragility suggests a less cooperative system with weaker intermolecular interactions.

(3) $$F=\frac{{BT}_{g,DRS}}{\ln(10){\left(T_{g,DRS}-T_{0}\right)}^{2}}$$

As shown in Table 3, the fragility of the FRPCs is generally higher than that of neat PC, except for FRPC5. This suggests that molecular motions in the FRPCs become more cooperative, accompanied by stronger intermolecular interactions. This enhanced cooperativity is likely due to hydrogen bonding between PC and phosphorylated-PC, as confirmed by FTIR spectra, which promotes greater segmental interactions. These findings are consistent with previous studies on the dynamic fragility of poly(vinyl methyl ether) and poly(2-vinylpyridine) blends [26]. It has been proposed that as the degree of intermolecular coupling increases, the configurational entropy available to the system decreases. This results in an increase in both the height of the potential energy barrier per monomer unit and the size of the cooperatively rearranging domain (CRD), leading to an increase in the system fragility [27,28,29]. Therefore, the increased fragility observed in FRPC1, FRPC2, FRPC3, and FRPC4 is due to the larger CRD. This larger CRD results from bulky phosphorylated-PC groups attaching to the PC chains through hydrogen bonds, as all these blends exhibit a homogeneous phase. Generally, PC is a rigid polymer, and its fragility is primarily influenced by chain stiffness and the bulkiness of side groups [30].

**Figure 8:**
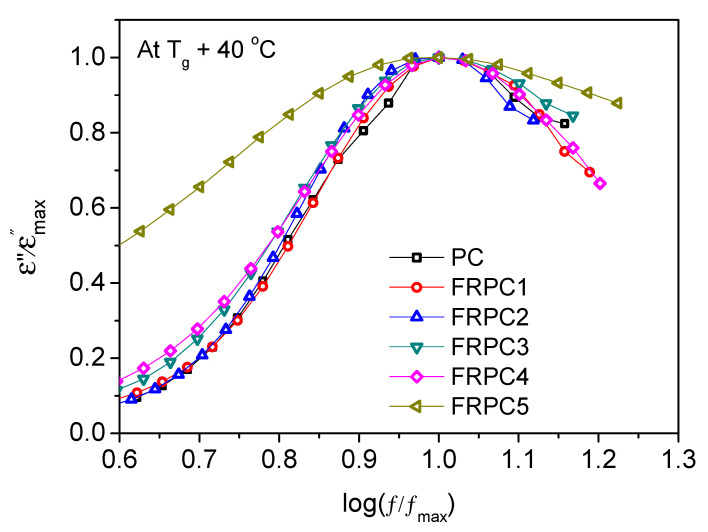
Normalize dielectric α processes of PC and FRPC at T_g_ + 40 °C.

In contrast, FRPC5 exhibits reduced fragility compared to neat PC, suggesting that the system is less cooperative. In these systems, molecular motions are less interdependent, and the intermolecular interactions between phosphorylated-PC and the PC chains are weaker. The dielectric α relaxation of PC and FRPCs was compared, as shown in Figure 8. The slight broadening at low frequencies observed for FRPC3 and FRPC4 is associated with hydrogen bonding, whereas the significant broadening of FRPC5 is likely due to constraints arising from the aggregation of phosphorylated polar groups. This aggregation, as confirmed by SAXS data, acts as physical crosslinks between the neat PC main chains, inhibiting segmental motion. This is reflected in the reduced dielectric loss of FRPC5 compared to the other FRPCs (Figure 6), which may contribute to its lower fragility compared to neat PC. These findings are consistent with our previous results on sulfonated polystyrene ionomers, where intermolecular cooperativity was not significantly affected by the physical crosslinks induced by the ion clusters [31]. The high-frequency broadening observed in FRPC5 is attributed to overlapping with local processes.

## 3. Materials and Methods

### 3.1. Materials and Reagents

Pellets of general-purpose PC grades (Makrolon 2405) were obtained from Bayer Material Science AG, Bangkok, Thailand. Methanol (MeOH), sodium hydroxide (NaOH), dichloromethane (CH_2_Cl_2_), and phosphoric acid (H_3_PO_4_) were purchased from QRёCTM, Auckland, New Zealand. Tetrahydrofuran (THF) and ethyl acetate (EtOAc) were obtained from Carlo Erba, Milan, Italy. Epichlorohydrin (ECH) was obtained from Acros organics, Geel, Belgium. Sodium sulfate (Na_2_SO_4_) and Dimethyl chlorophosphate were purchased from Loba Chemie (Maharashtra, India) and Sigma Aldrich, (Shanghai, China) respectively.

### 3.2. Preparation of Bisphenol-A from Polycarbonate (BPA-PC)

**Figure 9:**
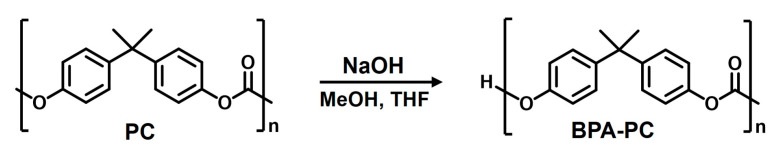
Methanolysis reaction of PC.

The methanolysis of polycarbonate (PC) was performed using a MeOH/THF mixture with a NaOH catalyst under moderate conditions, as shown in Figure 9 [19]. A 50 g sample of pure PC pellets, 50 mL of methanol (MeOH), 100 mL of tetrahydrofuran (THF), and 1 g of NaOH were added to a round-bottom flask equipped with a reflux condenser. The mixture was stirred at 40 °C for 35 min under atmospheric pressure. After the reaction, the solvent was evaporated using a rotary evaporator. The resulting product was a white solid, which was then ground and washed several times with distilled water to remove any residual NaOH. Finally, the product was dried in a vacuum oven at 90 °C overnight. Bisphenol A (BPA-PC) was obtained as a white solid with a yield of 90%.

### 3.3. Preparation of Phosphorylated-PC from BPA-PC

BPA-PC (0.05 mol) and epichlorohydrin (ECH) (0.15 mol) were added to a round-bottom flask. The mixture was heated to 90 °C, and then 15 mL of a 40% (*w*/*v*) NaOH solution was added. The mixture was then refluxed for 2–3 h. After the reaction, the mixture was diluted with ethyl acetate and washed with distilled water to remove NaCl. Anhydrous sodium sulfate was added to the product to remove any residual water. The solvent was evaporated using a rotary evaporator. After evaporation, a yellow viscous liquid of diglycidylether (Epoxy-PC) was obtained.

**Figure 10:**
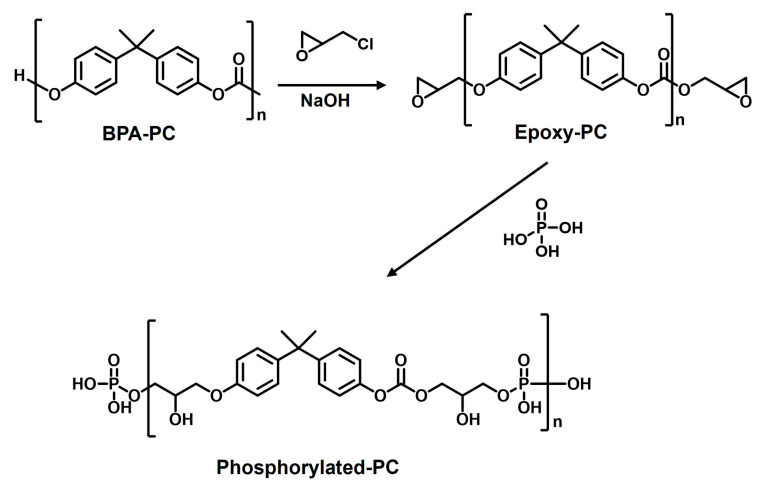
Synthesis methodology for phosphorylated-PC.

To prepare phosphorylated-PC (bisphenol-A-propyl bis(dihydroxy) phosphate), 0.04 mol of epoxy-PC was dissolved in 80 mL of ethyl acetate, and 0.08 mol of 85% phosphoric acid was added. The mixture was refluxed for 2 h, and the solvent was evaporated using a rotary evaporator at 80 °C. A yellow viscous liquid was obtained. The synthesis procedure is outlined in Figure 10. The viscosity of the phosphorylated-PC and PC solutions (10% *w*/*v* in THF) was measured using an AMETEK Brookfield DV2TLV viscometer, yielding values of 5.5 cP and 28.4 cP, respectively.

### 3.4. Preparation of Flame-Retardant PC Blends (FRPC)

Phosphorylated-PC was blended with PC at concentrations ranging from 1% to 5% *w*/*w*, and the resulting samples were labeled as FRPC1, FRPC2, FRPC3, FRPC4, and FRPC5, respectively. A PC and a phosphorylated-PC were dissolved in THF, poured into Petri dishes, and allowed to dry at room temperature. All solvent-cast samples were then compressed at a temperature range of 250–275 °C under a pressure of 20,000 lb, followed by water cooling for 10 min.

### 3.5. Characterizations

Attenuated total reflectance Fourier transform infrared spectroscopy (ATR-FTIR) was used to characterize the structure of the prepared phosphorus-containing flame-retardant PC. The analysis was performed using a Perkin Elmer System 2000 spectrometer, with signal averaging over 64 scans at a resolution of 2 cm^−1^.

Wide-angle X-ray diffraction (WAXD) patterns were acquired using a Scintag Pad V diffractometer with CuKα radiation (λ = 0.154 nm) at 35 kV and 30 mA. The sample films were scanned continuously at a rate of 1°/min over a 2θ range of 10° to 40°.

Small-angle X-ray scattering (SAXS) data were collected using a Molecular Metrology SAXS instrument with a CuKα radiation source (λ = 0.154 nm). A two-dimensional multi-wire detector captured the scattered X-rays, and the scattering vector (q) was calibrated using silver behenate as a standard. A parallel ionization detector was positioned in front of the sample to measure the incident and transmitted X-ray intensities, with the sample-detector distance set to 1.5 m. Data acquisition lasted 2 h. The resulting two-dimensional scattering data were azimuthally averaged to produce a one-dimensional intensity profile, I(q), as a function of the scattering vector q. The scattering vector, q, is defined as q = (4π/λ) sin θ, where λ is the X-ray wavelength and 2θ is the scattering angle.

The glass transition temperature was determined using a Seiko differential scanning calorimeter (DSC) 220 under N_2_ gas with a heating and cooling rate of 10 °C/min. The glass transition temperature (T_g_) shown in this paper was taken from the second scan of DSC thermograms.

The thermo-oxidative stability was assessed using a TA-Q500 thermogravimetric analyzer (TGA) under an O_2_ atmosphere, with a heating rate of 10 °C/min over a temperature range of 40–800 °C.

The flame retardancy was evaluated using vertical burning tests, performed according to the UL-94 test [23], with specimen dimensions of 125 × 125 × 0.2 ± 0.02 mm^3^. The weight change of each specimen before and after the test was recorded as weight loss (%), which is defined as the ratio of the weight change to the original weight of the specimen before the UL-94 test.

Dielectric relaxation spectroscopy (DRS) analyzes how charged species respond to an applied electric field. The motion of dipoles and ions in these charged species is influenced by the accessible frequency and temperature ranges. Dielectric constant (ε′) and dielectric loss (ε″) represent the real and imaginary parts of the complex dielectric constant, ε*, as shown in Equation 4. ε′ and ε″ were measured isothermally using a Novocontrol GmbH Concept 40 broadband dielectric spectrometer. Measurements were taken in the frequency range from 0.01 Hz to 1 MHz and across a temperature range of 0 to 200 °C. The temperature was maintained with a stability of ±0.5 °C. Sample films were placed between brass electrodes with a 10 mm diameter.

To extract the relaxation time τ_HN_ and dielectric relaxation strength Δε, which are related to the number of dipoles contributing to the dispersion, the isothermal dielectric loss ε″(*f*) curves were fitted using the Havriliak–Negami (HN) function. A combination of multiple HN functions and a DC loss contribution was employed to fit the experimental loss curves [32]:

(4) $$\varepsilon^{*}(\omega )=\varepsilon ’(\omega )-i\varepsilon ”(\omega )=\varepsilon_{\infty }-i\frac{\sigma_{0}}{(\varepsilon_{0}\omega {)}^{s}}+\sum \frac{\Delta \varepsilon }{[1+(i\tau_{HN}\omega {)}^{m}{]}^{n}}$$

where ε*, ε′, and ε″ are the complex, real and imaginary components of the dielectric permittivity, respectively; the dielectric relaxation strength Δε = ε_∞_ − ε_s_, where ε_∞_ and ε_s_ are the dielectric constants at limiting high and low frequencies, respectively; *σ*_0_ (= *σ*_dc_) is the dc conductivity (S/cm); ω is the angular frequency; τ_HN_ is the characteristic relaxation time; and m and n are shape parameters, indicative of the breadth of the relaxation and peak asymmetry, respectively. The exponent s characterizes the conduction process. The frequency maxima *f*_max_ can then be calculated from Equation 5 [24]:

(5) $$f_{max\;}=\frac{1}{2\pi \tau_{HN}}{\left[\frac{\sin\left(\frac{m\pi }{2+2n}\right)}{\sin\left(\frac{mn\pi }{2+2n}\right)}\right]}^{1/m}$$

## 4. Conclusions

The flame retardancy of PC was successfully enhanced by blending it with phosphorylated-PC. When the phosphorylated-PC content exceeds 4% by weight, nanoclusters of phosphorylated polar groups, approximately 14 nm in size, are formed. All FRPC blends remained amorphous and miscible. As the phosphorylated-PC content increased, the blends exhibited a decrease in the single glass transition temperature. The thermal stability of the FRPCs was enhanced compared to neat PC, with the char residue increasing as the phosphorylated-PC content rose. Flame retardancy was improved when the phosphorylated-PC content exceeded 3% by weight. Dielectric relaxation spectroscopy (DRS) revealed that adding phosphorylated-PC to neat PC induced a well-defined local (β) relaxation, which was absent in neat PC. This finding supports the presence of hydrogen bonding between the components of the blend, as observed in FTIR spectra. The β relaxation was attributed to the motion of the phosphorylated branches, which became more pronounced with increasing phosphorylated-PC content. All samples also exhibited the segmental (α) relaxation of the PC chains above the glass transition temperature. The size of the cooperatively rearranging domain plays a significant role in the dynamic fragility of the rigid FRPCs, particularly when the phosphorylated-PC content is less than 5% by weight. DRS analysis helped clarify the nanophase separation in FRPC5, which is attributed to the phosphorylated polar groups.

## Figures and Tables

**Table 1:** Mass of volatile products and char residue of PC and FRPCs.

Sample	Mass of Volatile Product (%)			Char Residue (%)
	450 °C	500 °C	550 °C	650 °C
PC	26.3	61.6	95.6	0.9
FRPC1	11.3	35.0	80.7	1.7
FRPC2	10.0	31.6	78.6	1.7
FRPC3	15.8	34.3	72.5	1.7
FRPC4	18.9	36.2	72.0	2.1
FRPC5	23.8	43.3	77.5	2.3

**Table 2:** UL-94 rating for PC and FRPCs.

Sample	UL-94 Rating	Dripping	Weight Loss (%)
PC	V-2	yes	2.8
FRPC1	V-2	yes	1.2
FRPC2	V-2	yes	0.7
FRPC3	V-2	yes	0.6
FRPC4	V-1	no	0.3
FRPC5	V-1	no	0.2

**Table 3:** T_g_s, VFT fit parameters, and fragilities for PC and FRPCs.

			VFT Parameters		
	T_g,DSC_	T_g,DRS_	B	T_0_	Fragility
Sample	(°C)	(°C)	(K)	(°C)	°
PC	145	144	2019	85	105
FRPC1	143	144	1815	91	117
FRPC2	140	141	1702	91	122
FRPC3	138	142	1707	92	123
FRPC4	135	141	1707	91	123
FRPC5	132	134	2285	67	90

## Data Availability

The data supporting these conclusions of the article are available upon request.
